# Vagus Nerve Stimulation as a Potential Adjuvant to Rehabilitation for Post-stroke Motor Speech Disorders

**DOI:** 10.3389/fnins.2021.715928

**Published:** 2021-08-19

**Authors:** Robert A. Morrison, Seth A. Hays, Michael P. Kilgard

**Affiliations:** ^1^School of Behavioral and Brain Sciences, University of Texas at Dallas, Richardson, TX, United States; ^2^Texas Biomedical Device Center, University of Texas at Dallas, Richardson, TX, United States; ^3^Erik Jonsson School of Engineering and Computer Science, University of Texas at Dallas, Richardson, TX, United States

**Keywords:** vagus nerve stimulation, motor speech disorder, stroke, speech therapy, dysphagia, neuroplasticity, rehabilitation

## Abstract

Stroke often leaves lasting impairments affecting orofacial function. While speech therapy is able to enhance function after stroke, many patients see only modest improvements after treatment. This partial restoration of function after rehabilitation suggests that there is a need for further intervention. Rehabilitative strategies that augment the effects of traditional speech therapy hold promise to yield greater efficacy and reduce disability associated with motor speech disorders. Recent studies demonstrate that brief bursts of vagus nerve stimulation (VNS) can facilitate the benefits of rehabilitative interventions. VNS paired with upper limb rehabilitation enhances recovery of upper limb function in patients with chronic stroke. Animal studies reveal that these improvements are driven by VNS-dependent synaptic plasticity in motor networks. Moreover, preclinical evidence demonstrates that a similar strategy of pairing VNS can promote synaptic reorganization in orofacial networks. Building on these findings, we postulate that VNS-directed orofacial plasticity could target post-stroke motor speech disorders. Here, we outline the rationale for pairing VNS with traditional speech therapy to enhance recovery in the context of stroke of speech motor function. We also explore similar treatments that aim to enhance synaptic plasticity during speech therapy, and how VNS differs from these existing therapeutic strategies. Based on this evidence, we posit that VNS-paired speech therapy shows promise as a means of enhancing recovery after post-stroke motor speech disorders. Continued development is necessary to comprehensively establish and optimize this approach, which has the potential to increase quality of life for the many individuals suffering with these common impairments.

## Introduction

Impairments affecting orofacial function are some of the most common lasting deficits after ischemic stroke, second only to hemiparesis. Roughly one third of those who undergo a stroke develop a speech impairment and nearly half will experience dysphagia (Laska et al., 2001; Engelter et al., 2006; Permsirivanich et al., 2009; Flowers et al., 2016; Stipancic et al., 2019). Acquired apraxia of speech, the inability to plan movements needed for normal speech production, dysarthria, reduced muscular coordination of speech, and dysphagia, or disrupted swallowing, can have devastating effects on quality of life. Stroke patients with orofacial impairments are twice as likely to require admittance to long-term care facilities (Martino et al., 2005; Smithard et al., 2006; Mitchell et al., 2020). Thus, the development of interventions to improve speech and reduce disability after stroke are of clear clinical importance.

A diverse array of speech-language therapies is used to treat motor speech disorders. Therapy may employ one or several rehabilitative strategies targeting rate and intensity of speech, prosody, and qualities affected by improper muscle control such as phonation and resonance. Course of treatment is commonly assessed based on the patient’s individual needs, and depending on severity of injury the clinician may choose to emphasize weak abilities to build strength or focus on coping strategies to circumvent particular deficits. While speech therapy is able to enhance function after stroke, many patients see only modest improvements after treatment (Langhorne et al., 2011; Mitchell et al., 2017), similar to other post-stroke motor rehabilitations (Dobkin, 2004). This partial restoration of function after rehabilitation suggests that there is a need for further intervention. Rehabilitative strategies that augment the effects of traditional speech therapy hold promise in reducing the disability associated with motor speech disorders, possibly enhancing recovery further (Ludlow et al., 2008). Here, we outline the rationale for pairing vagus nerve stimulation (VNS) with traditional speech therapy to enhance synaptic plasticity and improve recovery from post-stroke motor speech disorders.

## Plasticity Underlies Functional Improvements of Motor Speech Control Recovery

Neuroplasticity allows the brain to reorganize speech circuits disrupted by stroke and is a driving force behind recovery from motor speech disorders (Hartwigsen and Saur, 2019; Wilson and Schneck, 2021). Though speech production is heavily lateralized to the left hemisphere, increases in neuroplasticity during speech therapy can shift speech processing toward the right hemisphere (Anglade et al., 2014; Wan et al., 2014). Moreover, areas directly adjacent to the site of injury can undergo reorganization after therapy (Barritt and Smithard, 2009; Schlaug et al., 2009; Fridriksson et al., 2012). Plasticity in orofacial motor areas have been implicated in increases in function after speech therapy as well (Ludlow et al., 2008; Avivi-Arber et al., 2010), suggesting that plasticity of circuits directly involved in speech production plays a significant role in mediating recovery. These increases in neuroplasticity are thought to aid in the bypassing of injured circuits contributing to motor speech impairment, allowing the nervous system to compensate for loss of function (Khedr and Abo-Elfetoh, 2010; Kumar et al., 2011; Thiel et al., 2013). Given the importance of neuroplasticity underlying speech therapy and the incomplete recovery many patients experience after undergoing treatment, it is reasonable to conclude that interventions that further enhance the neuroplasticity produced by speech therapy could lead to greater functional outcomes.

## Vagus Nerve Stimulation

Vagus nerve stimulation has emerged as a method of enhancing rehabilitative outcomes for a wide range of neurological injuries, including stroke (Khodaparast et al., 2013, 2014; Hays et al., 2014b, 2016; Dawson et al., 2016, 2021; Pruitt et al., 2016, 2021; Ganzer et al., 2018; Kimberley et al., 2018; Meyers et al., 2018; Table 1). VNS increases the effects of rehabilitation through targeted enhancement of synaptic plasticity in central networks after injury. Electrical stimulation of the vagus nerve immediately enhances neuromodulatory function. Bursts of VNS rapidly activate the noradrenergic locus coeruleus (LC) and cholinergic nucleus basalis (NB), two major neuromodulatory centers in the brain (Dorr, 2006; Roosevelt et al., 2006; Nichols et al., 2011; Hulsey et al., 2016, 2017, 2019). Coincident release of these pro-plasticity neuromodulators coupled with neural activity related to rehabilitation facilitates synaptic plasticity in task-specific activated circuits (Dorr, 2006; Roosevelt et al., 2006; Seol et al., 2007; He et al., 2015; Hulsey et al., 2017).

**TABLE 1:**
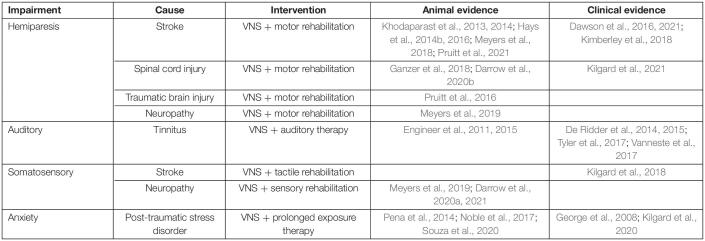
Vagus nerve stimulation (VNS) enhances a wide range of rehabilitative therapies.

## Vagus Nerve Stimulation Enhances Plasticity and Recovery in Motor Dysfunction

Vagus nerve stimulation enhances cortical representations related to a variety of motor activities. Stimulation of the vagus nerve paired with movement during motor training increases synaptic plasticity in activated circuits, selectively expanding cortical representations of the muscles active at the time of stimulation (Porter et al., 2012; Hulsey et al., 2016, 2019; Meyers et al., 2018; Morrison et al., 2019; Tseng et al., 2020). VNS-mediated synaptic plasticity also takes place in sub-cortical structures throughout task-related circuits (Ganzer et al., 2018; Borland et al., 2019). This targeted-enhancement of plasticity has proven useful in augmenting the effects of motor rehabilitation (Table 1). VNS-paired stroke upper limb rehabilitation significantly enhances motor recovery compared to traditional rehabilitation alone in rats (Khodaparast et al., 2013, 2016; Hays et al., 2014a,b, 2016). Furthermore, three clinical trials have now demonstrated that VNS-paired stroke rehabilitation significantly enhances functional recovery in humans, as indicated by increases in common clinical measures of upper limb motor function, including the Upper Extremity Fugl–Meyer Assessment and the Wolf Motor Function Test (Dawson et al., 2016, 2021; Kimberley et al., 2018).

The timing and electrical parameters of VNS appear to be of particular importance. VNS is able to potentiate circuits activated within a roughly 2 s window after stimulation occurs (Ganzer et al., 2018), meaning stimulation is most effective when paired coincident with or immediately after movements during rehabilitation. Electrical parameters of stimulation are also a critical determinant of the magnitude of VNS effects. Short half second bursts of 0.8 mA, 30 Hz stimulation with a 100 μs pulse width appear to be optimal for VNS-mediated synaptic plasticity and enhancement of recovery, and deviations from this stimulation paradigm often lessen or abolish VNS-mediated effects (Buell et al., 2018; Loerwald et al., 2018; Morrison et al., 2020, 2021; Pruitt et al., 2021).

## Vagus Nerve Stimulation Implantation, Safety, and Tolerability

Vagus nerve stimulation is an Frenchay Dysarthria Assessment (FDA)-approved therapy used to decrease symptoms of treatment resistant epilepsy and depression. VNS implantation consists of a pulse generator implanted near the clavicle, and two leads deliver electrical stimulation to a nerve cuff implanted in the neck around the left cervical branch of the vagus nerve. VNS devices have been implanted in over 1,00,000 people worldwide, and VNS is generally regarded as a safe and well-tolerated treatment (Yang and Phi, 2019).

The most common side effects arising from device implantation are acute vocal cord paresis (1% of patients) and acute lower facial weakness (1% of patients) (Ben-Menachem et al., 2015). As with any invasive procedure, risk of infection is also possible (3–6% of patients), and in some cases, these events result in device removal (Ben-Menachem et al., 2015; Yang and Phi, 2019). There have been recent efforts to minimize invasiveness by wirelessly powering the implanted nerve cuff, foregoing the use of an implanted pulse generator (Sivaji et al., 2019; Kilgard et al., 2020, 2021), which could conceivably decrease risk of infection in the future. Adverse events (AEs) arising from stimulation of the vagus nerve can include acute voice alteration, cough, dyspnea, paresthesia, headache, and neck pain (Ben-Menachem et al., 2015), however, these symptoms are most often present during the initial period of stimulation and decline over time (Morris and Mueller, 1999). It is also noteworthy that the duty cycle and cumulative amount of stimulation patients traditionally receive for treatment of epilepsy and depression is far greater than that of patients undergoing VNS-therapy paired with rehabilitation (Dawson et al., 2016, 2021; Kimberley et al., 2018), which could significantly decreases the magnitude or prevalence of AEs arising from stimulation.

Based on the known side effect profile of VNS, it is possible that some AEs associated with VNS, including acute voice alteration, cough, and similar side effects, could interfere with speech therapy. However, some preventative measures could be taken to minimize risk of this interference. First, because adverse effects of related to stimulation normally resolve over time, any effects that disrupt speech therapy during the beginning stages of treatment may subside as treatment progresses. Second, for stimulation-related AEs that prove too disruptive early in speech therapy may be circumvented through habituation. VNS has been shown to treat stroke in chronic stages, years after initial injury and onset of deficits (Dawson et al., 2016, 2021; Kimberley et al., 2018). Because the start of VNS therapy does not appear to be time sensitive, it is possible that patients with newly implanted devices could be habituated for a number of weeks to stimulation before speech therapy begins, allowing for the minimization of any AEs.

## Adapting VNS Therapy for Upper Limb Dysfunction to Treat Post-Stroke Motor Speech Disorders

Vagus nerve stimulation paired with upper limb rehabilitation enhances upper limb function after stroke (Dawson et al., 2016, 2021; Kimberley et al., 2018). Given the commonalities between limb motor control and speech motor control rehabilitation, it has been suggested that principles of motor learning and rehabilitation often applied to upper limb treatment could be applied to speech therapy as well (Ludlow et al., 2008; Maas et al., 2008; Grimme et al., 2011). Recent work demonstrates that VNS can significantly enhance synaptic plasticity in corticobulbar circuits mediating orofacial movement. Repeatedly pairing VNS with jaw movement increases the area of motor cortex that evokes jaw movements via intracortical microstimulation (Morrison et al., 2020, 2021). Because VNS enhances recovery from upper limb dysfunction by increasing synaptic plasticity in corticospinal upper limb circuits, enhancement of speech therapy for post-stroke motor speech disorders such as apraxia and dysarthria could similarly be realized via VNS-mediated plasticity in corticobulbar circuits involved in speech production. This possibility is further supported by the fact that plasticity in cortical orofacial areas is already implicated in recovery from motor speech dysfunction (Ludlow et al., 2008; Avivi-Arber et al., 2010), suggesting VNS could prove a useful adjuvant to enhance the effects of various traditional speech therapy interventions after stroke.

While there are certainly commonalties between speech motor control and limb motor control rehabilitation, there are significant differences between orofacial and limb biomechanics, their anatomical networks, and the neural activity governing their movement. Speech motor acts have large, variable degrees of freedom due to their complexity, and compared to motor acts of the limbs, have much faster rates of production (Grimme et al., 2011). Speech production is complex even compared to upper limb movements and is governed by a diverse array of orofacial and laryngeal muscles that are even further modulated by changes in respiration and airflow (Kent, 2004). Though speech motor acts and upper limb movements may vary in anatomy and complexity, it is possible that VNS could overcome these differences due to its known ability to potentiate and reorganize circuits in a diverse array of networks. For example, VNS-paired training can direct plasticity in cortical and subcortical auditory networks related to sound perception (Borland et al., 2019), hippocampal and amygdala networks related to memory and anxiety (Zuo et al., 2007; Pena et al., 2014), at multiple points along the corticospinal pathway mediating limb movement (Ganzer et al., 2018), and in corticobulbar pathways mediating jaw movement (Morrison et al., 2020). That VNS can enhance plasticity in such a diverse array of systems suggests it could possibly potentiate speech networks in a similar manner.

**FIGURE 1:**
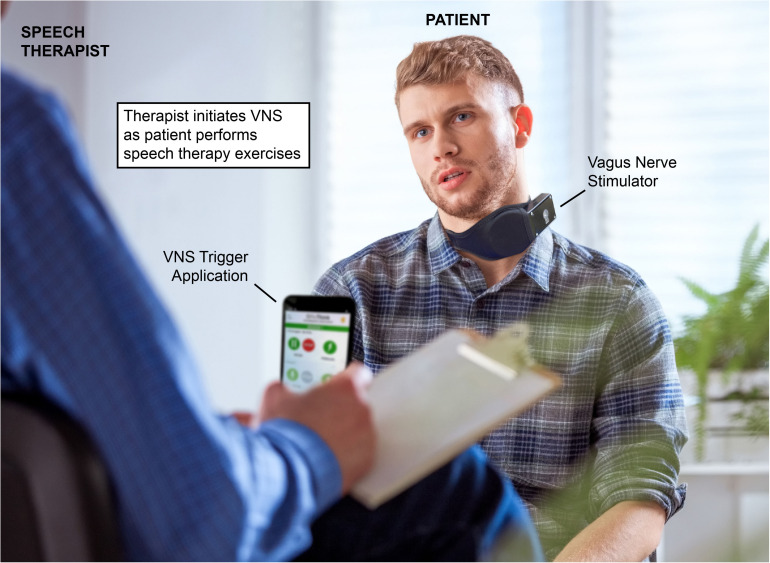
Example of vagus nerve stimulation (VNS) paired speech therapy. A patient with a VNS implant performs speech therapy exercises. The speech therapist initiates VNS via a mobile application at moments the therapist deems conducive to the patient’s recovery. The mobile device used by the therapist activates the power control module, which wirelessly powers the implanted vagal nerve cuff. Reproduced with permission from www.istockphoto.com.

Using a paradigm similar to that of existing VNS-paired upper limb stroke rehabilitation (Dawson et al., 2016; Kimberley et al., 2018) could allow for pairing of VNS with multiple speech therapy techniques. VNS paired with specific exercises could allow for targeted enhancement of orofacial circuits involved in specific deficits, enhancing recovery. Under this rehabilitation paradigm, the therapist leading the speech therapy session would activate the patient’s VNS implant via a wireless remote (Figure 1). The therapist would conduct speech therapy normally, triggering VNS when the patient is performing speech therapy exercises, emphasizing moments they view as conducive to recovery. This timed, performance-dependent application of VNS strives to reorganize and strengthen the circuits activated during speech therapy that mediate recovery, enhancing the effects of rehabilitation. Clinical efficacy of VNS-paired speech therapy could be quantitatively determined using a mix of deficit-specific assessments such as the Apraxia of Speech Rating Scale (ASRS; Strand et al., 2014), the FDA-2 (Enderby, 1980), and quality of life observations using activity of daily living (ADL) and the Barthal ADL Index (Geusgens et al., 2006). Using VNS, it is possible that rehabilitative exercises that are already evidenced to promote recovery could be further enhanced, such as dysarthria-specific oromotor exercises (Mackenzie et al., 2014), articulatory feedback training for apraxia of speech (Katz and McNeil, 2010; Ballard et al., 2015), and respiratory muscle training for those with post-stroke respiratory weakness (Menezes et al., 2016).

## Vagus Nerve Stimulation as an Alternative to Previously Studied Neuromodulatory Strategies in Speech Therapy

Increasing neuroplasticity during speech therapy to enhance its effects has long been considered a promising treatment. Pharmacological augmentation of speech therapy has been proposed as a means of increasing the magnitude of neuroplasticity during treatment to gain better recovery outcomes (Berthier, 2005). However, clinical investigation of speech therapy paired with a wide range of drugs affecting neuromodulatory systems has generally shown mixed effects on recovery (McNeil et al., 1997; Noble and Benfield, 1998; Berthier, 2005), further complicating the use of pharmacologically augmented speech therapy. While a number of these drugs of interest may enhance neuroplasticity, many have contraindications, particularly in those with underlying cardiovascular issues that can contribute to stroke. Many of these drugs activate neuromodulatory systems similar to those activated by VNS, but one critical difference is the timing of neuromodulation. The systemic nature of drugs do not lend to potentiation of circuits contributing to recovery from speech disorders, but instead, lead to a global, sustained activation of neuromodulatory systems. Alternatively, VNS-paired rehabilitation accounts for this lack of temporal specificity by only increasing neuromodulator levels transiently, allowing for the targeting of specific neural circuits mediating recovery.

Another approach to the problem of temporal specificity in neuromodulatory activity, transcranial direct current stimulation (tDCS; Fridriksson et al., 2011; Hamilton et al., 2011; Doeltgen et al., 2015; Elsner et al., 2015; Turkeltaub et al., 2016) and transcranial magnetic stimulation (TMS; Hamilton et al., 2011; Naeser et al., 2012) have been used in conjunction with speech therapy with more promising, yet still mixed outcomes. While timed bursts of tDCS and TMS may solve for the lack of temporal specificity seen in pharmacological augmentation of speech therapy, these treatments locally activate glutamatergic and GABAergic neurons (Medeiros et al., 2012; Cirillo et al., 2017), which may actively interfere with circuits mediating motor function. VNS, however, only increases neuromodulatory function and does not interfere with ongoing neural spiking (Hulsey et al., 2016, 2017, 2019; Morrison et al., 2019, 2020). The temporal specificity of VNS positions it as a promising alternative to drugs, tDCS, and TMS in treating motor speech dysfunction after stroke.

## Applications for VNS-Paired Treatment of Dysphagia

Post-stroke dysphagia is another commonly experienced disability, affecting approximately 50–75% of patients (Mann et al., 1999; Martino et al., 2005; Singh, 2006; Barritt and Smithard, 2009; Stipancic et al., 2019). While post-stroke dysphagia is often acute, resolving within a month after injury, up to 40% of patients can still display disrupted swallowing a year after onset (Terré and Mearin, 2009). Chronic dysphagia increases risk for aspiration pneumonia, admittance to long-term care facilities, and death (Martino et al., 2005; Smithard et al., 2006). While behavioral mitigation strategies and diets limiting food consistency are common treatment prescriptions, these are often ineffective at improving long-term outcomes (Carnaby et al., 2006). Similar to recovery from apraxia of speech, plasticity in orofacial motor areas in cortex appears to be a determinant of recovery of function in post-stroke dysphagia. After stroke, increases in oropharyngeal representation in the contralesional hemisphere accompany recovery from dysphagia (Hamdy et al., 1998; Barritt and Smithard, 2009). Given the high comorbidity of post-stroke speech apraxia and dysphagia, their similarities in underlying pathologies, and their overlap in therapeutic strategies, VNS may prove an effective adjuvant to dysphagia treatment, such as oromotor exercises as well.

## Conclusion

Vagus nerve stimulation has emerged as a method of enhancing rehabilitative outcomes for a wide range of neurological disorders. Here, we suggest pairing VNS with traditional speech therapy to enhance recovery from post-stroke speech motor dysfunction. We outline clinical success of VNS-paired physical rehabilitation after stroke, which demonstrates that VNS can induce plasticity in task-activated motor systems, enhancing patient recovery outcomes. Furthermore, we summarize the observations that VNS can enhance plasticity in orofacial networks when paired with jaw movement, which supports its use as a potential adjuvant to speech therapy in treating motor speech dysfunction. Based on this evidence, we believe VNS-paired speech therapy shows promise as a means of enhancing recovery after post-stroke motor speech disorders, and future study of this new treatment has potential to increase function, and subsequently quality of life for the many suffers of these common conditions.

## Author Contributions

RM drafted the manuscript. All authors contributed to the manuscript revision and approved the submitted version.

## Conflict of Interest

MK has a financial interest in MicroTransponder Inc., which is developing VNS for stroke. The remaining authors declare that the research was conducted in the absence of any commercial or financial relationships that could be construed as a potential conflict of interest.

## Publisher’s Note

All claims expressed in this article are solely those of the authors and do not necessarily represent those of their affiliated organizations, or those of the publisher, the editors and the reviewers. Any product that may be evaluated in this article, or claim that may be made by its manufacturer, is not guaranteed or endorsed by the publisher.
